# Affordability of health care under publicly subsidized insurance after Massachusetts health care reform: a qualitative study of safety net patients

**DOI:** 10.1186/s12939-015-0240-5

**Published:** 2015-10-29

**Authors:** Leah Zallman, Rachel Nardin, Monica Malowney, Assaad Sayah, Danny McCormick

**Affiliations:** Department of Medicine, Cambridge Health Alliance, Cambridge, MA 02139 USA; Institute for Community Health, Malden, MA USA; Harvard Medical School, Boston, MA USA; Department of Population Health, Maimonides Medical Center, Bronx, NY USA; Cambridge Health Alliance, Cambridge, MA USA

## Abstract

**Introduction:**

The Affordable Care Act (ACA) and the 2006 Massachusetts (MA) health reform law, on which the ACA was based, aimed to improve the affordability of care largely by expanding publicly sponsored insurances. Both laws also aimed to promote consumer understanding of how to acquire, maintain and use these public plans. A prior study found an association between the level of cost-sharing required in these plans and the affordability of care. Preparatory to a quantitative study we conducted this qualitative study that aimed to examine (1) whether cost sharing levels built into the public insurance types that formed the backbone of the MA health reform led to unaffordability of care and if so, (2) how insurances with higher cost sharing levels led to unaffordability of care in this context.

**Methods:**

We interviewed 12 consumers obtaining the most commonly obtained insurances under MA health reform (Medicaid and Commonwealth Care) at a safety net hospital emergency department. We purposefully interviewed a stratified sample of higher and low cost sharing recipients. We used a combination of inductive and deductive codes to analyze the data according to degree of cost-sharing required by different insurance types.

**Results:**

We found that higher cost sharing plans led to unaffordability of care, as evidenced by unmet medical needs, difficulty affording basic non-medical needs due to expenditures on medical care, and reliance on non-insurance resources to pay for care. Participants described two principal mechanisms by which higher cost sharing led to unaffordability of care: (1) cost sharing above what their incomes allowed and (2) poor understanding of how to effectively acquire, maintain and utilize insurance new public plans.

**Conclusions:**

Further efforts to investigate the relationship between perceived affordability of care and understanding of insurance for the insurance types obtained under MA health reform may be warranted. A potential focus for further work may be quantitative investigation of how the level of calibration of cost-sharing to income and understanding of insurances under the MA reform was associated with perceived affordability of care.

**Electronic supplementary material:**

The online version of this article (doi:10.1186/s12939-015-0240-5) contains supplementary material, which is available to authorized users.

## Introduction

The Affordable Care Act (ACA), modeled closely on the Massachusetts (MA) health reform law of 2006, expands coverage to the uninsured largely through a Medicaid expansion and offering subsidized insurance to low and middle income individuals though newly created health insurance exchanges. Both health reform laws contained provisions intended to improve consumer understanding of insurance plan options and their benefit features, such as services covered and the degree and type of cost-sharing. Under MA health reform, MA devoted significant resources to hire outreach workers, run telephone helplines and provide grants to community groups to provide outreach [1, 2]; under the ACA, additional resources to support insurance navigators, certified application counselors, and insurance exchanges were provided [3]. Additional file 1: Appendix 1 summarizes the process for obtaining publicly subsidized insurance. Early evidence from the ACA which began implementation in 2014 demonstrates that consumers’ ability to compare benefits and premiums improved during the first three months of implementation of insurance exchanges; however many reported challenges in plan selection [4]. Limited data is available on consumer understanding of insurance after MA health reform [5], and no prior studies focus on the consumers receiving publicly subsidized plans, which represent the majority of the newly insured [6].

In addition to devoting resources to improving understanding of insurance, both reform laws devote resources to improving the affordability of care. Under MA health reform, in addition to expanding Medicaid to residents with incomes below 138 % of the federal poverty level (FPL), the state devoted financial resources to providing subsidized plans, called Commonwealth Care (CWC), for low to middle income populations [6]. There were three types of CWC, which differed by income eligibility and cost-sharing requirements; Additional file 1: Appendix 2 demonstrates the cost-sharing requirements for these plans. Residents with incomes below 100 % FPL were eligible for CWC type 1, which is fully premium-subsidized insurance with low cost-sharing similar to Medicaid – that is no premiums and only small copays for medications. Residents with incomes 100–300 % FPL were eligible for CWC types 2 or 3, which required monthly premiums and had higher and more complex cost-sharing. Under its implementation of the ACA in 2014, MA has committed to subsidizing plans (called ‘ConnectorCare’) that have similar costs and coverage requirements as CWC [7]. These efforts led to overall improvements in perceived affordability of care after MA health reform [8–10]. However, evidence suggests that affordability remained challenging for some populations [5, 11], in particular socio-economically disadvantaged populations [10–12]. Similarly, early evidence points to improved affordability of care after the ACA, though difficulty affording care remains [13].

Prior studies have demonstrated that for low income individuals, higher cost-sharing often leads to forgone or delayed care [14–16]. The ACA’s Medicaid expansion and benefit design of subsidized plans were developed with the objective of improving affordability of care for low to middle income persons. Like the MA reform, the subsidized plans under the ACA have differing levels of cost-sharing based on income [17]. One prior study found an association between the level of cost-sharing required in the publicly subsidized plans offered under MA reform and the affordability of care [11]; because this study used cross-sectional data, it could not establish a causal link between cost-sharing level and affordability of care and did not explore the mechanisms by which this association may occur. Thus, no evidence exists on how cost-sharing to income ratios built into ACA- mandated subsidized plans may affect affordability of care.

We conducted this study preliminary to a larger quantitative study about the role that cost sharing and knowledge about insurance play in affordability of care in the context of the health reforms in Massachusetts. The objectives of the current study were to examine (1) whether different cost sharing levels built into the public insurance types that formed the backbone of the MA health reform led to unaffordability of care and if so, (2) how insurances with higher cost sharing levels led to unaffordability of care – specifically, did high cost sharing requirements in relationship to income and did poor understanding of how to acquire and utilize insurance lead to unaffordable care? In order to achieve these aims, we conducted in-depth open-ended interviews with a total of 12 low to middle income consumers who received Medicaid (called “MassHealth” in MA) or CWC obtained under the MA health reform in 2013.

## Methods

### Study conceptual framework

We examined themes that pertained to plan members’ perceived affordability according to degree of plan cost-sharing. Formal quantitative definitions of affordability (usually defined by an arbitrary proportion of income spent on healthcare) require information on health care spending but such information is often difficult for individuals to recall accurately. Thus, like other investigations of affordability often do [9, 11], this study examined perceived affordability of care, or the respondents’ subjective assessment of whether care was affordable to them. For our analyses, we combined patients with Medicaid and CWC type 1 (low cost-sharing), and those with CWC type 2 and 3 (higher cost-sharing), given the similarities in their cost-sharing features (Additional file 1: Appendix 2). Although premiums and cost sharing differed slightly between CWC type 2 and 3 such that a middle cost sharing group (CWC type 2, which required lower copayments and premiums than CWC type 3) could have been examined, we were unable to examine this group separately due to limited financial resources available for performing this study. This study was approved by the Institutional Review Board at Cambridge Health Alliance.

### Sample selection and recruitment

We conducted interviews of patients presenting for emergency care in one emergency department at the second-largest safety-net hospital in Massachusetts. Because we were interested in perspectives of those with low and higher cost sharing, we purposefully selected a stratified sample of 6 low and 6 higher cost sharing plan recipients. This study was conducted to inform the conceptual framework and design of a larger quantitative survey study; the sample size of the current study was limited due to limited financial resources available. Within each cost sharing stratum, we selected a convenience sample. Trained research assistants identified eligible patients and verified their insurance using the New England Health Exchange Network [18], a statewide database that is updated daily. Participants were eligible if they were aged 18–64, spoke English, had an Emergency Severity Index [ESI] [19] of 2–5 (we excluded those with an ESI of 1, the most severely ill), were insured by Medicaid or a CWC plan for at least 6 months, and had visited a doctor at least once during that time. During business hours (weekdays between 9 am and 5 pm), research assistants identified potential participants and approached patients in their examination rooms after confirming with the clinical care team that recruitment would not impact clinical care. The research assistant conducted a detailed informed consent process with each potential study participant in which potential harms and benefits of participation were discussed and all questions were answered by the research assistant. Those wishing to participate provided verbal informed consent. Eighteen potential participants were identified and 12 (six participants from each cost-sharing group) were recruited. Six potential participants were not included: five declined due to feeling too ill and one was not invited to participate because the interviewer was her primary care physician. No participation incentives were offered. Table 1 provides a profile of the participants.

### Data collection

We developed an interview guide based on a review of the relevant literature [5, 10–12, 20] and experiences of two of the study investigators who practice primary care at the study hospital. Additional file 1: Appendix 3 shows the interview guide. Domains included perceptions of affordability of care, understanding of health insurance, and experiences acquiring and maintaining insurance. Interviews were conducted after full implementation of MA health reform (2008) and prior to full implementation of the ACA (2014), during October and November of 2013, by two authors (DM and LZ) lasted 15–30 min, and were audio-recorded and transcribed.

**Table 1:** Characteristics of Participants (N=12)

	N
Female (N)	6
Hispanic (N)*	1
Race (N)*	
White	6
Black	2
Other	2
Insurance (N)	
Higher cost-sharing (Commonwealth Care 2 and 3)	6
Low cost-sharing (Medicaid and Commonwealth Care 1)	6
	Median (range)
Age	36 (23-56)
Number of monthly medications	2.5 (0-8)
Number of doctors visits, past year	3 (1-12)

*missing data on two participants

### Analysis

We entered data into the qualitative data analysis program Dedoose. We developed a coding structure that included a combination of deductive and inductive codes. Deductive codes were based on concepts that we explicitly asked participants about (e.g., understanding of insurance, ability to afford care, ability to afford non-medical basic needs). Inductive codes were based on major ideas that emerged from participants’ responses (e.g., reliance on non-insurance based resources). This strategy allowed us to examine our study questions (i.e., through deductive codes) while allowing us to capture other key themes that surfaced from the data (i.e., through inductive codes). A trained research manager (MM) and a study investigator (LZ) read the initial interviews and developed a draft code book, which was then modified after they applied the code book to an additional set of interviews. Discrepancies were resolved by consensus. Subsequently, the trained research manager conducted all coding and coding was discussed at regular coding meetings.

## Results

### High cost sharing led to unaffordability of care

Higher cost sharing plan participants described their plans as leading to difficulty affording care. Difficulty affording medical care was evidenced by higher cost sharing plan participants’ descriptions of their inability to get needed medical care due to cost, inability to afford other basic needs due to paying for medical care, and the need to rely on non-insurance based resources in order to pay for medical care. Low cost-sharing participants mainly described difficulties obtaining needed medical care due to cost, while higher cost-sharing participants also reported inability to afford other basic needs.

#### Inability to get needed medical care due to cost

Participants with both low and higher cost-sharing plans described difficulty obtaining needed medical care due to costs. However, difficulty obtaining medical care was less common among low cost-sharing participants; in fact, most low cost-sharing plan enrollees reported no difficulty affording their care. When difficulty affording care occurred among low cost-sharing participants, the problems were of smaller magnitude: “I’ve had to delay a day or two getting meds. It only happened once so far” and “[the] only time it affects me is if I’m out of work . . . Other than that, it’s no problem”.

On the other hand, higher cost-sharing plan participants described avoiding medications and doctor’s visits. For some of these participants, avoiding paying for medications was a regular event “I stretch my medication to two months sometimes, every other day, sometimes every 3 days. . . Every time I go to refill my medication it’s like $60 or $70”. Although the highest copayment for generic medications for these insurance types is $12.50 per prescription (Additional file 1: Appendix 2), multiple medications and/or specialty high cost medications may have led to higher total copayments for some participants. Avoidance also applied to doctor’s visits: “When I go to visit the doctor, half the price [would be] good. Little things, I don’t go running to the doctor. I don’t even do [an annual] physical [exam]. [It] has to be really bad”. Perceptions of ability to afford medical care were sensitive to increased costs for other basic needs; “Sometimes in the winter if my heating bill is a little too high, I won’t pick up my medication one month”. Others described making their own determination of which medications were essential to purchase, skipping those that seemed less essential. For both groups, forgoing medical care was associated with negative health consequences, particularly uncontrolled symptoms (i.e., persistent pain, worsening depression).

#### Inability to afford other basic needs

Higher cost-sharing plan participants described how inability to afford care was associated with inability to afford other basic needs, principally difficulty paying for rent and food, and being unable to return to college. When asked how medical expenses affected the ability to afford other items, a representative higher cost participant responded: “obviously food . . . I am cutting off food” and “Well, most of my money goes to prescriptions . . . Getting a place to live, forget it. That’s why I’m in a shelter”.

#### Reliance on non-insurance based resources

For both higher and low cost-sharing participants, inability to afford care was associated with needing to rely on other sources of support such as: loans from family members or friends, healthcare providers’ willingness to accept late payments and enrollment in other government programs. One participant described how other government services helped: “most of my money goes to prescriptions . . . Well, right now, I get food stamps so that does help”. This same participant described a pharmacy that provided medications despite his being unable to pay copayments; “Well, I pay $3.65 for each medication and it can strap me, but here’s the kicker (and I love this): they [the pharmacy] have a red book under the counter. If I can’t pay. . . they put the money in [the register] so I can cash out and they’ve been very gracious”. Other participants described the importance of having a physician who was willing to accept late payment and having family members who could loan money for copayments during lower income periods.

### How higher cost sharing plans led to unaffordable care

Participants described two principal underlying mechanisms by which higher cost sharing led to unaffordable care: (1) cost sharing above what their incomes allowed – that is, high costs in relationship to income, and (2) poor understanding on how to effectively acquire, maintain and utilize their publicly sponsored insurance.

#### High costs in relationship to income

Some respondents, particularly those with higher cost-sharing plans, identified the amount they paid for services and premiums as higher than they could afford. As one participant with the highest cost-sharing plan, CWC type 3, described: “I don’t make [enough] money that I can pay $100 for this [emergency room] visit, or $85 every month [for premiums]”. This was particularly true for those with more medical needs who reported difficulty affording care: “I have to go for eye, this allergies, this cold, this knee problem. So I get sick very often and I can’t afford [it]”.

### Effective Acquisition, Maintenance and Use of Insurance: the Importance of Knowledge

#### Difficulty acquiring and maintaining insurance

A dominant theme was difficulty in acquiring or maintaining insurance, or both; for respondents, this difficulty led to periods of inactive insurance and inability to afford care during these periods, a . A representative participant described the process of acquiring insurance as, “I won’t say easy. It was one step at a time, it really was perseverance”. Most described a disconnect between the knowledge that insurers expected them to have and what they actually understood: “It’s almost like you wanted some step-by-step ‘Insurance for Dummies’ [instructions] . . I think there was an assumption of [a] basic understanding”. Study participants insured by Medicaid (a low cost sharing plan obtained directly from the state) were more likely to describe relative ease in acquiring insurance than were those with CWC plans: “actually they [the Medicaid application procedures] were pretty straightforward”.

Maintaining insurance was particularly problematic for many participants. Participants were often not aware of a lapse in their insurance until they sought medical care, and often expressed confusion as to how the lapse occurred. As one participant with a higher cost sharing plan, CWC type 3, described: “They took me off the plan, I don’t know what happened. Maybe it expired. They [the emergency room] told me I’m not covered”. Several participants described miscommunication with insurance providers (e.g., not receiving letters or not realizing that they had to select a plan in order to activate their insurance) as the reason for lapses in their insurance. However, some described receiving but ignoring letters due to prioritizing other competing demands over insurance.

Difficulty acquiring and maintaining insurance was commonly associated with delays in care or inability to afford care. One participant articulated how lack of coverage led to a delay in care: “I couldn’t pick up prescriptions and I didn’t go to the doctor because I wasn’t covered”. Another commented: “there’s been times where I needed to go to the ER and I didn’t have coverage at the time”. Some participants sought emergency care despite lacking coverage, resulting in inability to afford care, “I had to [go to the emergency room]. They sent me bills and I literally would ignore the bills and think, hopefully when I reinstate my insurance, I’ll deal with it later. I don’t know, maybe there’s someone still out there chasing me for that 400 or 500 bucks”.

In order to cope with the difficulty acquiring and maintaining insurance, nearly all participants relied on other supports (usually someone at their healthcare institution, such as a social worker or insurance specialist, and occasionally friends or family) to navigate getting and keeping their insurance. As one Commonwealth Care type 1participant described, “I signed up for free care and it was very easy. They walked you through it down at registration, here at the hospital”. Several participants insured by Medicaid (MassHealth) described telephone helpline agents as particularly helpful, “MassHealth is, you always get a person. I called . . . and it made it pretty easy”.

### Lack of knowledge about how to use insurance in a cost-effective manner

A key factor determining affordability of care for both cost-sharing groups was knowledge about how to use insurance in a cost-effective manner. Knowledge of costs allowed participants to plan for health care expenditures (e.g., set aside money for medication copayments) and seek care in settings that were lower cost to them. As one participant noted, “I know [emergency room] visits are covered so was more than happy to get seen right away”. In contrast, some participants described avoiding emergency rooms because seeing a primary care physician would be less expensive. As one participant with the highest cost-sharing plan, CWC type 3, stated: “that’s why we went to primary care rather than going to emergency. This [has been] going on since yesterday but we thought it was gonna be more expensive, so we went to primary care [and were referred to the emergency room], so then we ended up paying $15 and now $100; we pay twice”.

On the other hand, for higher cost-sharing participants, uncertainty about costs led to seeking care in unaffordable settings, such as the emergency department. Several participants noted that they would have sought care elsewhere if they had known the cost of emergency care; as one participant with the highest cost-sharing plan (CWC type 3) stated: “Yeah, I wouldn’t come here if I know that [it would cost me $100]. I would go to minute clinic . . . I can’t afford it [in the emergency department]. I didn’t know”. Knowledge about when to call insurers allowed one participant to negotiate terms and request more affordable rates. This participant called her insurer when her financial situation changed and was able to get a lower cost-sharing plan: “my rent was going up but my salary was staying the same . . . so I called [the insurer], and I just told them what was happening . . . so that’s when they came in with the $3 [for medication copayments]”.

## Discussion

In this qualitative study of a small sample of patients with publicly subsidized insurances after MA health reform seeking emergency care at a safety net institution, we found that higher cost sharing led to difficulty affording care among recipients of the higher cost-sharing insurance types available under the MA reform; recipients of low cost-sharing insurance types described much less, though still some, difficulty affording care. Greater difficulty affording care among higher cost sharing plan participants was evidenced by greater inability to afford other basic needs, adverse medical consequences, and reliance on non-insurance based resources (such as loans from family). Participants’ lack of knowledge on how to use their insurance in a cost effective manner was associated with greater inability to afford care among both groups but was particularly prominent among higher cost-sharing plan participants. We also noted that this difficulty was more prominent among those obtaining insurance through the health insurance exchange (CWC plans 1, 2 and 3) than among those obtaining insurance directly from the state (Medicaid).

Our finding that level of cost-sharing among insurances that formed the backbone of MA health reform led to unaffordable care is consistent with prior literature about cost sharing in other contexts [14, 15] and advances understanding of the impacts of the specific costs sharing levels built in to the MA health reform [11]. This finding suggests that further investigation as to whether cost-sharing levels may have been calibrated to income in such as a way as to result in differing levels of perceived affordability of care under MA health reform may be warranted. One complicating factor is that eligibility for type of insurance – and therefore cost sharing level – is determined by level of income. Prior studies demonstrate that low income individuals have difficulty affording basic needs in general (not just health care) [21, 22]. Understanding the relationship between income, cost-sharing level and affordability of care are areas for further investigation.

Our study provides new information about how well one small convenience sample of Massachusetts residents understood publicly subsidized insurances available under MA health reform. We are aware of no published investigations of understanding of insurance among residents who obtained the publicly subsidized insurance types in this study, the most commonly acquired insurance types under MA health reform [6]. In addition, no prior study has examined associations between consumer understanding of plans and procedures for acquiring coverage, and affordability of care. Our study raises the possibility that consumer understanding of insurance plans may be associated with inability to afford care; that is, that difficulty understanding insurance plans may be linked to consumer perceptions that care is unaffordable. However, this is a preliminary study with a limited sample size and further investigations are needed in order to confirm and further understand the association between understanding of insurance and inability to afford care.

In addition, our findings suggest two other areas for further exploration. Our finding that Medicaid recipients described relative ease in acquiring and learning about insurance suggests that there may be lessons that can be learned from the Massachusetts Medicaid enrollment and education process. Similarly, some of our recipients reported seeking care in settings that may not have been the most cost-efficient to them or to the healthcare financing system – for example, seeking care in primary care when ultimately emergency care was needed (or vice versa). Further investigation about whether and how to guide consumers on where to seek care may be warranted, therefore.

Because our results derive from a small sample of patients seeking emergency care at one safety net institution, they may not be generalizable to other populations. By recruiting emergency department patients, however, we were able to accurately verify the insurance type, thus reducing the high rates of error associated with patient self-reported insurance type see in other studies [20]. We did not specifically inquire whether and to what degree participants interacted with programs for consumer education (such as insurance exchanges and helplines). We also cannot determine how often the experiences and perceptions described by study participants occur among other populations in Massachusetts without additional quantitative studies. Lastly, we did not collect data on the health impacts of experiencing difficulty affording care in this study. However, a substantial prior literature has documented the harmful effects of not being able to afford needed care. As an initial step the use of qualitative methodology allowed us to explore the relatively unknown experiences with and relationship between affordability of care, understanding of insurance and cost-sharing under Massachusetts health reform.

Our study suggests that even with Massachusetts health reform, which improved affordability of care for its residents [8–10] and devoted resources toward making insurance comprehensible for all of its residents [1, 2], difficulty affording care and understanding of insurance may have remained for some. Our results suggest that further efforts to investigate the relationship between understanding of insurance for insurances obtained under MA health reform and affordability of care may be warranted. They also suggest the need for further work, using large quantitative studies, to confirm our findings and to better understand how the level of calibration of cost-sharing to income under the MA and ACA reforms is with affordability of care; that is, whether the calibration for some plans was such that cost-sharing was high enough, relative to income, so as to cause some residents to perceive care to be unaffordable. Because the goal of both reforms was to make care affordable for low and moderate income individuals, high cost-sharing relative to income - to the extent that it is associated with perceived lack of affordable care - would be an important policy consideration.

## Additional file

Additional file 1: Appendix 1. Description of mechanism for obtaining publicly sponsored insurance. **Appendix 2.** Cost-Sharing and Health Benefits in Massachusetts Publicly Subsidized Health Insurance Plans in 2013. **Appendix 3.** Interview Guide. (DOC 56 kb)
