# Supplementary material for: Affordability of health care under publicly subsidized insurance after Massachusetts health care reform: a qualitative study of safety net patients
Source: Int J Equity Health. 2015 Oct 29;14:112. doi: 10.1186/s12939-015-0240-5 (PMC4625740; doi:10.1186/s12939-015-0240-5)
Supplement: Additional file 1: Appendix 1. — Description of mechanism for obtaining publicly sponsored insurance. Appendix 2. Cost-Sharing and Health Benefits in Massachusetts Publicly Subsidized Health Insurance Plans in 2013. Appendix 3. Interview Guide. (DOC 56 kb) [file 12939_2015_240_MOESM1_ESM.doc]

**Appendix 1. Description of mechanism for obtaining publicly sponsored insurance**

Under the MA reform (prior to the ACA implementation in MA), low to middle income individuals seeking insurance applied to the state Medicaid office by mail, in-person at a Masshealth Enrollment Center or, with the assistance (required) of a state trained councilor employed by a health care provider organization or community based organization via a state website called the Virtual Gateway. The MA Medicaid office then made a determination of eligibility for Medicaid or publicly subsidized insurance, called Commonwealth Care (CWC) plans offered exclusively through the state’s health insurance exchange, the “Connector”. This determination was based on applicant income, employment and immigration status. Individuals deemed eligible for Medicaid were sent a letter notifying the applicant of Medicaid eligibility and requesting the applicant to call the state Medicaid office by phone to choose between two types of Medicaid plans (“primary care physician” or “managed care”) about which information was provided. Once the applicant selected a plan, it was issued from the state Medicaid office.

Those deemed eligible for CWC were also sent a letter notifying the applicant of eligibility for one of 3 CWC plan types (1, 2 or 3) that differed in their benefit design and cost-sharing features. The letter requested the applicant to call the Connector customer service phone line or go to the Connector website to select a particular CWC plan carrier offering a plan type for which the applicant qualified (1, 2 or 3). Information on cost-sharing, covered services, and provider networks was available on the website and from the customer service phone line. Once a plan was selected, and where premium were required, paid for, a CWC plan was issued. Once insured, recipients had to maintain their insurance by providing eligibility information on an annual basis.

**Appendix 2.** Cost-Sharing and Health Benefits in Massachusetts Publicly Subsidized Health Insurance Plans in 2013

| Cost-Sharing | Publicly Subsidized Insurance Plan | | | |
| --- | --- | --- | --- | --- |
|  | Medicaid ^*^ | Commonwealth Care Plans^†^ | | |
|  |  | Type 1 | Type 2 | Type 3 |
| Copays (dollars) |  |  |  |  |
| Outpatient visit |  |  |  |  |
| Primary Care provider | 0 | 0 | 10 | 15 |
| Specialist | 0 | 0 | 18 | 22 |
| Inpatient care | 3 | 0 | 50 | 250 |
| Emergency care | 0 | 0 | 50 | 100 |
| Prescription drugs (30 days) |  |  |  |  |
| Generic drug | 1-3.65 | 1-3.65 | 10 | 12.50 |
| Drug on preferred list | 3.65 | 3.65 | 20 | 25 |
| Drug not on preferred list | NC | 3.65 | 40 | 50 |
| Imaging (CT, MRI, PET) |  | 0 | 30 | 60 |
| Vision (eye exam) | 0 | 0 | 10 | 20 |
| Rehabilitation services | 0 | 0 |  |  |
| Inpatient (Rehab Hospital) | 3 | 0 | 50 | 250 |
| Outpatient Visit | 0 | 0 | 10 | 20 |
| Maternity and family planning | 0 | 0 | 0 | 0 |
| Dental | 0 | 0 | NC | NC |
| Maximum copay for prescription | 250 | 250 | 400 | 650 |
| Maximum copay excluding prescriptions | 36 | 0 | 600 | 1200 |
| Premium (dollars/month) | 0^‡^ | 0 | 3-81 | 118-182 |
| Deductible (dollars) | 0^§^ | 0 | 0 | 0 |

Source: HealthConnector Website[7] and [Mass.Gov](http://Mass.Gov/) website.[6, 24]

NC=Service not covered

^*^ In Massachusetts there are seven separate Medicaid plans for those under 65 years of age. We report the covered benefits and cost-sharing for MassHealth Standard, MassHealth Basic, MassHealth Family Assistance, MassHealth CommonHealth and MassHealth Essential. MassHealth Limited and MassHealth Prenatal have very limited covered benefits.[6]

**Appendix 3. Interview Guide**

**Understanding of health insurance concepts**

1. Describe the health insurance you have. How does your health insurance work?

Can you describe how you use it/what it covers?

1. Do you pay a monthly fee? What is that for/what it is it called?
2. Do you pay more of your expenses at the start of your insurance year? Why is that? What is that called?
3. Do you pay when you go to the doctor or get medications? How does that payment relate to your health insurance? What is it for? What is it called?
4. [if uncertainty elicited in answers above] It sounds like you have some uncertainty about these concepts. Tell me about a time when this uncertainty has affected your ability to get care.

**Emergency Room**

1. Do you have another place like a doctor’s office or clinic where you get care?
2. Tell me about why you came to the emergency today instead of going to a doctor or clinic.
   1. Was cost a factor in why you came to the emergency room? How so? Why not?

**Premiums**

Some people pay a certain amount each month to keep their insurance active, called a premium.

1. Do you currently pay a premium or have you paid one in the past?
2. Tell me about how your premiums have affected your ability to get health care?
3. How have premiums affected your ability to pay other bills such as food/heat/rent? How so?

**Copays**

Some people pay a certain amount each time they see the doctor or get a medication, called a copay.

1. Do you currently pay a doctor or medication copay or have you paid one in the past?
2. Tell me about how your copays have affected your ability to get health care?
3. Have you had to delay or forgone seeing the doctor or getting a medication because you couldn’t pay the visit copay? Tell me about that . .
4. How have copays affected your ability to pay other bills such as food/heat/rent?

***Acquiring and Maintaining Insurance***

1. Tell me about the time when you signed up for your current insurance. How did that go? What was it like?
2. What have your experiences been with trying to stay on your insurance in the past?
3. Have you had times when you were not covered by your health insurance?
   1. If yes, what could have prevented that? Tell me about the last time . . what lead to that? How did that affect your ability to get care?
   2. If no, what has prevented you from having lapses? Has there been a person or system that has helped you? How has this person or system helped you?
